# Prevalence of suicide attempts and correlates among first-episode and untreated major depressive disorder patients with comorbid dyslipidemia of different ages of onset in a Chinese Han population: a large cross-sectional study

**DOI:** 10.1186/s12888-022-04511-z

**Published:** 2023-01-04

**Authors:** Yang Jiang, Yaoyao Lu, Yi Cai, Chengjiang Liu, Xiang-Yang Zhang

**Affiliations:** 1 Guangxi Zhuang Autonomous Region Brain Hospital, Guangxi Liuzhou, 545005 China; 2 Department of Psychosomatic Disorders, Shenzhen Mental Health Center/Shenzhen KangNing Hospital, Shenzhen, 518000 Guangdong China; 3 Department of General Medicine, Anhui Medical University, He Fei, 230601 China; 4 CAS Key Laboratory of Mental Health, Institute of Psychology, Chinese Academy of Sciences, Beijing, 100101 China

**Keywords:** Major depressive disorder, Dyslipidemia, Suicide attempts, Age of onset

## Abstract

**Background:**

Patients with dyslipidemia are at increased risk for suicide, especially those with major depressive disorder (MDD). Few studies have investigated the independent effects of suicide attempts on comorbid dyslipidemia in patients with MDD. Moreover, there are no comparisons of differences in factors associated with suicide attempts among patients with MDD with dyslipidemia at different ages of onset. The aim of this study was to investigate the prevalence of suicide attempts and associated variables in first episode and untreated patients with MDD with comorbid dyslipidemia at different ages of onset.

**Methods:**

We recruited 1718 patients with first-episode untreated MDD in this study. Demographical and clinical data were collected, and lipid profiles, thyroid function, and blood glucose levels were measured. The Hamilton Depression Scale 17 (HAMD-17), Hamilton Anxiety Scale (HAMA), Clinical Global Impression Severity Scale (CGI), and Positive and Negative Syndrome Scale (PANSS) positive subscale were assessed for depression, anxiety and illness severity, as well as psychotic symptoms, respectively.

**Results:**

The percentage of patients with MDD with comorbid dyslipidemia was 61% (1048/1718). Among patients with MDD with comorbid dyslipidemia, the incidence of suicide attempts was 22.2% (170/765) for early adulthood onset and 26.5% (75/283) for mid-adulthood onset. Independent factors associated with suicide attempts in early adulthood onset patients with MDD with dyslipidemia were as follows: HAMA score (B = 0.328, *P* < 0.0001, OR = 1.388), Suspicion /persecution (B = -0.554, *P* = 0.006, OR = 0.575), CGI (B = 0.878, *P* < 0.0001, OR = 2.406), systolic blood pressure (B = 0.048, *P* = 0.004, OR = 1.049), hallucinatory behavior (B = 0.334, *P* = 0.025, OR = 1.397), and TPOAb (B = 0.003, *p* < 0.0001, OR = 1.003). Independent factors associated with suicide attempts in mid-adulthood onset patients with MDD with comorbid dyslipidemia were as follows: HAMA score (B = 0.182, *P* < 0.0001, OR = 1.200), CGI (B = 1.022, P < 0.0001, OR = 2.778), and TPOAb (B = 0.002, *P* = 0.009, OR = 1.002).

**Conclusion:**

Our findings suggest an elevated risk of suicide attempts in patients with MDD with comorbid dyslipidemia. The incidence of suicide attempts was similar in the early- and mid-adulthood onset subgroups among patients with MDD with dyslipidemia, but the factors associated with suicide attempts were different in these two subgroups.

## Background

The relationship between blood lipids and major depressive disorder（MDD） has attracted widespread attention. Several surveys have found that 52.3% of patients with MDD have hypertriglyceridemia and 44.7% have hypercholesterolemia [1]. Previous studies have indicated that individuals with MDD have lower levels of serum high-density lipoprotein cholesterol (HDL) than healthy controls [2]. One study found has found higher low-density lipoprotein (LDL) cholesterol and a higher LDL/HDL ratio, but lower HDL, in depressed patients than in healthy controls [3].Several studies have shown that metabolic disturbances (low HDL, hypertriglyceridemia, and hyperglycemia) in people with chronic depression who were taking antidepressants [4]. In another study, walking or bicycling excursions lowered LDL cholesterol, serum triglyceride, and total cholesterol, raised HDL levels, and alleviated depressive symptoms in patients with MDD, suggesting that lipid levels are linked to the severity of depression [5].

MDD is the psychiatric disorder most closely associated with suicide. A meta-analysis has reported that 11.1% of depressed patients commit suicide in their lifetime [6]. Approximately one million individuals worldwide choose to die by suicide each year, and roughly half of those who die by suicide can be diagnosed as MDD [7]. According to reports, the suicide rate in the United States is about 85.3 per 100,000 people with depression [8]. Some reports indicate that the annual suicide rate for patients with MDD in the Chinese population is 100 per 100,000 people [9]. Patients with undiagnosed and untreated depression have a very high risk of suicide, especially those with comorbidities (e.g., anxiety and dyslipidemia) and adverse life events [10].

There is growing evidence that blood lipids are associated not only with depression but also with suicide attempts [11, 12]. Several studies have shown that low levels of Total cholesterol (TC) and LDL could predict suicidal behavior in individuals with MDD [13]. A previous study noted that suicide attempters who experienced MDD had lower serum triglycerides [14]. Other studies have suggested that low lipid metabolism may be a biological marker for assessing suicide risk [15]. Other studies suggest that TC may be a biomarker of suicide risk in patients with MDD [16]. However, the relationship between blood lipids and suicide is also controversial. Several recent studies have found no links between triglyceride levels, LDL, total cholesterol, and suicide attempts after controlling for suicide diagnosis and method [17]. According to a meta-analysis, cholesterol levels in patients with MDD who attempted suicide and those who did not attempt suicide were similar to healthy controls [18]. However, in China, no studies have examined whether the incidence of suicide attempts is higher in patients with MDD with dyslipidemia compared with those without dyslipidemia.

Patients with MDD at different ages of onset have different clinical features. According to the Sequential Treatment Alternatives for Depression Relief (STAR*D) study, MDD women whose symptoms began age 18 had a longer duration of disease, longer current episodes, more frequent episodes, higher suicidal tendencies, and more severe symptoms [19]. Previous studies have shown that pre-adult-onset patients with MDD have more suicidal tendencies than adult-onset patients with MDD [20]. Results from a study of Chinese patients with MDD showed that these early-onset patients were more likely to feel lonely and unemployed, more likely to have atypical psychotic depressive episodes, and more likely to have features of bipolar disorder compared with late-onset patients [21]. However, the differences in the incidence of suicide attempts and their associated factors among patients with MDD with dyslipidemia at different ages of onset are unclear.

Therefore, the purposes of our current study were 1) to explore the differences in suicide attempts between patients with MDD with and without dyslipidemia; and 2) to determine the incidence of suicide attempts and associated factors in patients with MDD with and without dyslipidemia at different ages of onset.

## Methods

### Participants

This was a cross-sectional study designed and conducted by the Department of Psychiatry of the First Hospital of Shanxi Medical University. Eligible patients were recruited from 2015 to 2017. The inclusion criteria applied in this study were as follows: (1) MDD diagnosis confirmed by two clinically experienced psychiatrists according to the Structured Clinical Interview for DSM-IV (SCID); (2) age between 18 and 60 years; (3) HAMD-17 score greater than or equal to 24; (4) first episode without any previous medication or psychotherapy; and (5) Han Chinese ethnicity. The following exclusion criteria were established: (1) other Axis I disorders other than MDD; (2) comorbid severe physical illnesses; (4) substance and drug abuse or dependence, except for nicotine; (5) pregnant or breastfeeding women; (6) those patients with bipolarity currently diagnosed by two research psychiatrists based on SCID, as well as those with a history of bipolar disorder; (7) those patients on lipid-lowering medications or any other medications that could have impacted their lipid metabolism.

This study was approved by the Institutional Review Board of the First Hospital of Shanxi Medical University. The patients meeting criteria for inclusion and exclusion signed an informed consent form and were allowed to withdraw at any time from this study voluntarily.

The sample size was determined by the use of the formula, *n* = Z^2^p(1-p)/d^2^. *n* = number of sample size; Z = 95% confidence interval equal to 1.96; d = 0.05 (5%), marginal error; *p* = expected prevalence, equal to 0.2014 according to a previous study [22]. This provided an estimate for the 247 patients in the sample. Out of a total of 1796 screened individuals, only those patients who satisfied the inclusion and exclusion criteria were enrolled in our study. 78 patients were excluded for the following reasons: (1) They were a pregnant or breastfeeding (*n* = 10). (2) They had a substance use disorder (*n* = 9), a severe personality disorder (*n* = 15), a severe physical illness (*n* = 9), refused to participate in the study (*n* = 21), were unable to be interviewed due to an acute clinical condition (*n* = 5), and had other unspecified reasons (*n* = 9). Finally, the sample size to be included in our study was 1718, which was significantly larger than the required sample size (*n* = 247), indicating that our sample size had sufficient power.

### Clinical measurements

Professionally trained researchers used self-designed questionnaires to collect socio-demographic data on participants, including marital status, age, sex, education, duration of disease, and age at onset of MDD. We also collected patients' systolic and diastolic blood pressure, height, and weight, and calculated their body mass index (BMI). The research nurse measured systolic and diastolic blood pressures in the participant's left arm using an Omron Hem digital monitor with the participant seated in a quiet position. According to previous studies [23, 24] 45 years was used as the early-adulthood onset and mid-adulthood onset for MDD. Patients were classified as early-adulthood onset when their onset was younger than 45 years of age, and mid-adulthood onset when their onset were older than or equal to 45 years of age.

The HAMD-17, HAMA, PANSS positive subscale, and CGI were used to assess depression, anxiety, and psychotic symptoms, and illness severity, respectively. Patients with a total score of 24 on the HAMD have severe depressive symptoms [25]. The 17-item HAMD scale had reliability coefficients between 0.88 to 0.99 and validity coefficients of 0.94 or higher [26]. The HAMA has 14 items on a 5-point scale from 0 (no symptoms) to 4 (very severe symptoms) [27]. The total HAMA score reflects the severity of the patient's anxiety symptoms. It is the measure of anxiety that is most often used in the study of depression [28]. Positive symptoms were evaluated using the PANSS positive subscale. Patients were considered to have psychotic symptoms when the positive symptom subscale had a score of 15 or above. The PANSS scale had reliability coefficients of 0.73 to 0.83 and validity coefficients of 0.77 to 0.89 [29]. Prior to the start of the study, these psychiatrists received clinical research training in the assessment of the PANSS, HAMD, HAMA, and CGI. The inter-rater correlation coefficient for HAMD, HAMA and PANSS total scores between the two psychiatrists were 0.85, 0.84 and 0.82, respectively.

All patients with MDD were interviewed face-to-face in a quiet setting in terms of their suicide attempts. Based on previous research on suicide [30], all patients were asked: "Have you attempted suicide in your lifetime?" If patients answered "yes," they were labeled as suicide attempters, and if they answered "no," they were labeled as non-suicide attempters. For those suicide attempters, we continued to ask them about the number of suicide attempts, the manner of each attempt, and the approximate date of attempted suicide.

### Physical and biochemical parameters measurements

Blood samples were collected from all participants between 6 and 8 a.m. after an overnight fast. Then blood samples were immediately transported to the laboratory center at the hospital to measure the following biomarker parameters: blood glucose, TC, triglycerides (TG), HDL, and LDL, as well as thyroid stimulating hormone (TSH), free triiodothyronine 3 (FT3), free thyroxine 4 (FT4), thyroid peroxidase antibody (TPOAb), and anti-thyroglobulin (TgAb).

The definition of dyslipidemia was based on the third report of the National Cholesterol Education Program (NCEP) Expert Panel on Detection, Evaluation, and Treatment of High Blood Cholesterol in Adults' final report (NCEP-ATP III) [31]. We also referenced the 2016 Chinese guidelines for the prevention and treatment of dyslipidemia in adults [32]. Dyslipidemia was defined as a combination of TG, LDL-C, and TC expressed as ≥ 2.3, ≥ 3.4, and ≥ 6.2 mmol/L, respectively.

### Statistical analysis

We used SPSS 25.0 for statistical analysis. We used chi-square tests to examine categorical variables. When continuous variables conformed to a normal distribution, analysis of variance (ANOVA) was used. When continuous variables did not conform to a normal distribution, the Mann–Whitney U test was used. We conducted univariate analyses to identify the differences in variables related to suicide attempts in individuals who have MDD with dyslipidemia of early adult onset and mid-adulthood onset. We used Bonferroni correction for multiple comparisons. Those significantly different variables between these groups with and without a history of suicide attempts were then entered into a logistic regression analysis (backward: Wald) to examine the variables for suicide attempts. Furthermore, we used the area under the receiver operating characteristic (AUCROC) to identify key parameters in the ability to distinguish between individuals with MDD comorbid dyslipidemia who attempted suicide and those who did not. A consistency statistic between 0.7 and 0.8 was considered usually acceptable. We used *P* < 0.05 (two-tailed) as the level of significance.

## Results

### Prevalence of suicide attempts among patients with MDD with comorbid dyslipidemia by age of onset.

The prevalence of comorbid dyslipidemia in patients with MDD was 61% (1048/1718). Furthermore, the incidence of suicide attempts was 23.4% (245/1048) in patients with MDD with comorbid dyslipidemia, which was higher compared with MDD without dyslipidemia (101/670, 15.1%; 2 = 17.52, *P* < 0.001, OR = 1.42, 95% CI: 1.19–1.69). In addition, among patients with MDD with comorbid dyslipidemia, the incidence of suicide attempts was 22.2% (170/765) for early adult onset and 26.5% (75/283) for mid-adulthood onset, without significant difference (χ2 = 2.112, *P* = 0.162). Among patients with MDD without dyslipidemia, the incidence of suicide attempts was 14.6% (72/493) for early adult onset and 16.3% (29/177) for mid-adulthood onset, without significant difference (χ2 = 0.322, *P* = 0.570).

### Clinical characteristics and biochemical indicators of suicide attempters and non-attempters in dyslipidemia patients with MDD with early-adulthood onset and mid-adulthood onset.

Table 1 shows that among early-adulthood onset patients with MDD with dyslipidemia, suicide attempters had higher PANSS positive symptom, suspicion/persecution, CGI, hallucinatory behavior, CGI, HAMD and HAMA scores, longer duration of disease, higher systolic and diastolic blood pressure (BP), as well as high serum levels of TPOAb, TC, LDL-C, TSH, and TgAb, but lower HDL levels compared with non-suicide attempters (all *P* < 0. 01). All these significances passed Bonferroni correction (all corrected *p* < 0.05), except for duration of disease (Bonferroni corrected *p* > 0.05).

**Table 1:** Socio-demographics and clinical characteristics in patients with and without suicide attempts in early adulthood onset

Variable	MDD comorbid Dyslipidemia		*F/Z/x*^*2*^	P
	With SA (*N* = 170)	Without SA (*N* = 595)		
Age	29.71 ± 8.11	28.71 ± 8.71	1.40	0.162
Illness duration, months	6.44 ± 4.44	5.51 ± 3.87	2.64	0.008
Age of onset, years	28.51 ± 8.01	28.56 ± 8.56	1.33	0.183
HAMD-17	32.46 ± 2.68	30.43 ± 2.68	8.75	0.000
HAMA	23.71 ± 3.27	20.37 ± 3.06	12.37	0.000
Psychotic positive score	11.28 ± 6.07	8.42 ± 3.43	5.88	0.000
Hallucinatory behavior	1.82 ± 1.56	1.21 ± 0.82	4.91	0.000
Systolic BP, mmHg	123.75 ± 11.01	116.54 ± 9.54	8.39	0.000
Diastolic BP, mmHg	78.78 ± 6.77	75.31 ± 6.03	6.02	0.000
TSH,,uIU/mL	7.31 ± 2.63	5.10 ± 2.34	10.57	0.000
FT3, pmol/L	4.95 ± 0.77	4.96 ± 0.74	0.18	0.856
FT4, pmol/L	17.18 ± 3.29	16.77 ± 3.03	1.51	0.130
TgAb, IU/L	154.10 ± 314.58	75.72 ± 209.52	3.06	0.002
TPOAb, IU/L	173.69 ± 284.08	51.20 ± 122.58	5.47	0.000
TC, mmol/L	6.16 ± 1.03	5.44 ± 1.10	7.62	0.000
TG, mmol/L	2.62 ± 0.99	2.61 ± 1.02	0.11	0.910
HDL, mmol/L	1.08 ± 0.29	1.22 ± 0.29	5.41	0.000
LDL, mmol/	3.44 ± 0.93	3.14 ± 0.92	3.67	0.000
CGI	6.57 ± 0.64	5.95 ± 0.72	10.14	0.000
BMI, kg/m2	24.47 ± 2.45	24.50 ± 1.83	0.14	0.884
Suspiciousness/Persecution	2.12 ± 1.40	1.46 ± 0.91	5.80	0.000
Gender			1.04	0.310
Male, n (%)	57(33.5%)	225(37.8%)		
Female, n (%)	113(66.5%)	370(62.2%)		
Marital status			0.113	0.737
Single, n (%)	65(38.2%)	236(39.7%)		
Married, n (%)	105(61.8%)	359(60.4%)		

*HAMD*-17 Hamilton rating Scale for Depression-17 items, *HAMA* Hamilton anxiety rating Scale, *BMI* Body mass index, *BP* Blood pressure, *TC* Total cholesterol, *TG* Triglycerides, *HDL* High-density lipoprotein cholesterol, *LDL* Low-density lipoprotein cholesterol, *FBG* Fasting blood glucose, *TSH* Thyroid stimulating hormone, *FT3* Free triiodothyronine, *FT4* Free thyroxine

As shown in Table 2, among mid-adulthood onset patients with MDD with dyslipidemia, suicide attempters had higher PANSS positive symptom, CGI, HAMD, and HAMA scores, higher systolic and diastolic blood pressure, as well as high serum levels of TPOAb, TC, LDL, TSH, and TgAb, but lower HDL levels compared with non-suicide attempters (all *P* < 0. 01). All these significances passed Bonferroni correction (all corrected *p* < 0.05), except for diastolic blood pressure (Bonferroni corrected *p* > 0.05).

**Table 2:** Socio-demographics and clinical characteristics in patients with and without suicide attempts in mid-adulthood onset

Variable	MDD comorbid Dyslipidemia		*F/Z/x*^*2*^	P
	With SA (*N* = 75)	Without SA (*N* = 208)		
Age	51.89 ± 4.05	50.69 ± 4.39	2.07	0.039
Illness duration, months	8.47 ± 5.61	8.25 ± 5.71	0.28	0.780
Age of onset, years	50.53 ± 4.07	50.35 ± 4.37	2.05	0.041
HAMD	32.52 ± 2.94	30.82 ± 2.65	4.61	0.000
HAMA	24.05 ± 3.96	20.83 ± 3.08	7.18	0.000
Psychotic positive score	14.00 ± 7.69	8.84 ± 4.09	5.53	0.000
Hallucinatory behavior	2.53 ± 1.95	1.33 ± 1.01	5.12	0.000
Systolic BP, mmHg	131.65 ± 9.94	127.54 ± 6.83	3.31	0.001
Diastolic BP, mmHg	80.48 ± 9.39	77.36 ± 6.90	2.63	0.010
TSH,,uIU/mL	7.74 ± 2.73	5.61 ± 2.32	6.49	0.000
FT3, pmol/L	4.96 ± 0.59	4.83 ± 0.71	1.403	0.162
FT4, pmol/L	16.19 ± 2.72	16.75 ± 3.21	1.34	0.181
TgAb, IU/L	209.72 ± 393.38	96.27 ± 337.77	2.38	0.018
TPOAb, IU/L	158.97 ± 231.51	71.83 ± 161.69	3.54	0.000
TC, mmol/L	6.21 ± 0.96	5.69 ± 1.06	3.68	0.000
TG, mmol/L	2.60 ± 1.04	2.50 ± 0.95	0.78	0.441
HDL-C, mmol/L	1.11 ± 0.27	1.16 ± 0.31	1.14	0.253
LDL-C, mmol/	3.57 ± 0.77	3.34 ± 0.87	2.04	0.042
CGI	6.67 ± 0.60	6.01 ± 0.76	6.75	0.000
BMI, kg/m2	24.58 ± 1.85	24.56 ± 1.83	0.09	0.929
Suspiciousness/Persecution	2.53 ± 1.95	1.33 ± 1.01	6.76	0.000
Gender			1.37	0.240
Male, n (%)	24(32%)	52(25%)		
Female, n (%)	51(68%)	156(75%)		
Marital status			0.223	0.640
Single, n (%)	3(4%)	6(2.8%)		
Married, n (%)	72(96%)	202(97.2%)		

*HAMD*-17 Hamilton rating Scale for Depression-17 items, *HAMA* Hamilton anxiety rating Scale, *BMI* Body mass index, *BP* Blood pressure, *TC* Total cholesterol, *TG* Triglycerides, *HDL* High-density lipoprotein cholesterol, *LDL* Low-density lipoprotein cholesterol, *FBG* Fasting blood glucose, *TSH* Thyroid stimulating hormone, *FT3* Free triiodothyronine, *FT4* Free thyroxine

### Factors associated with suicide attempts in early-adulthood onset and mid-adulthood onset MDD with dyslipidemia

**Fig. 1:**
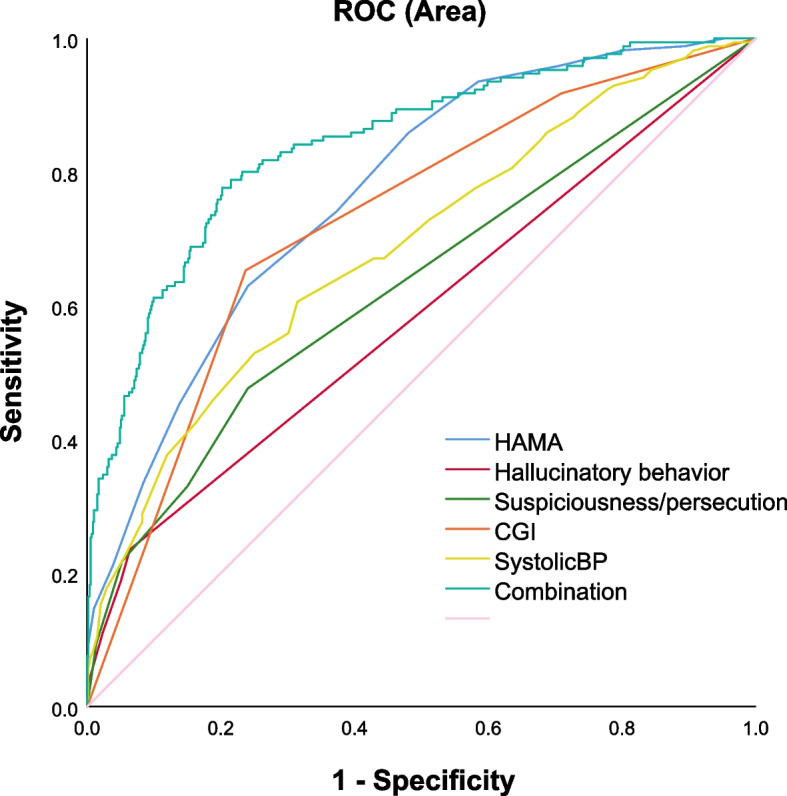
The discriminatory capacity of related factors for patients with suicide attempts in early adulthood onset. The area under the curve of the HAMA score, hallucinatory behavior, suspiciousness/persecution, CGI score, systolic BP, and the combination of these three factors were 0.77, 0.59, 0.63, 0.73, 0.69, and 0.84, respectively

We entered variables with significant differences in univariate analysis into logistic regression (backward: Wald) to find factors associated with suicide attempts in patients with MDD with dyslipidemia at different ages of onset. Table 3 shows the following correlates of suicide attempts in dyslipidemia patients with MDD with early-adulthood onset: HAMA score (B = 0.182, *P* < 0.0001, OR = 1.200), CGI (B = 1.022, *P* < 0.0001, OR = 2.778), and TPOAb (B = 0.002, *P* = 0.009, OR = 1.002). In addition, AUCROC showed the following values for each risk factor: 0.77 for HAMA, 0.59 for hallucinatory behavior, 0.63 for suspicion/persecution, 0.73 for CGI, and 0.69 for systolic blood pressure. Finally, the combination of HAMA, hallucinatory behavior, suspicion/persecution, and CGI was ≥ 0.8 (Fig. 1).

**Table 3:** Factors associated with suicide attempts in patients with comorbid dyslipidemia in early adulthood onset

	B	Wald statistic	P	OR	95%CI
HAMA score	0.328	44.34	0.000	1.388	1.260–1.530
Suspicion/Persecution	1.815	7.52	0.006	1.739	1.170–2.630
CGI	0.878	20.01	0.000	2.406	1.638–3.530
Systolic BP	0.048	8.13	0.004	1.049	1.010–1.080
Hallucinatory behavior	0.334	5.01	0.025	1.397	1.040–1.870
TPOAb	0.003	20.18	0.000	1.003	1.002–1.005

*HAMA* Hamilton anxiety rating scale, *HAMD* Hamilton rating scale for depression, *BP* Blood pressure, *CGI* Clinical global impression, *TPOAb* Thyroid peroxidases antibody

**Fig. 2:**
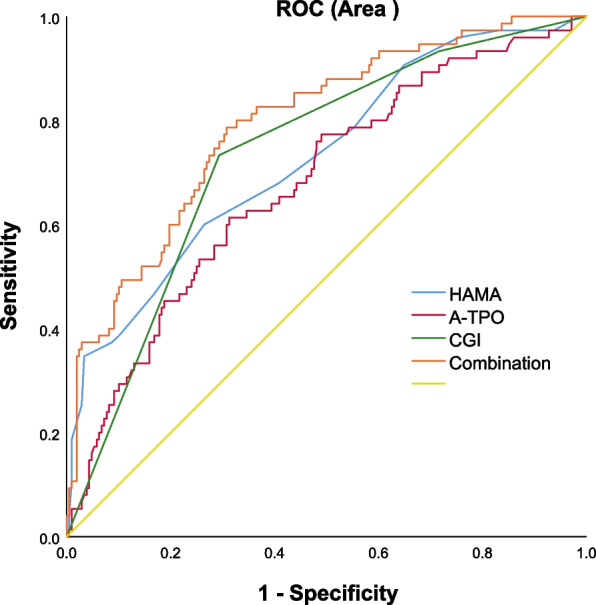
The discriminatory capacity of related factors for patients with suicide attempts in mid-adulthood onset. The area under the curve for the HAMA score, TPOAB score, and CGI score, as well as their combination, were 0.73, 0.68, and 0.79, respectively

Table 4 shows the following correlates of suicide attempts in dyslipidemia patients with MDD with mid-adulthood onset: HAMA score (B = 0.182, *p* < 0.0001, OR = 1.200), CGI (B = 1.022, *p* < 0.0001, OR = 2.778), and TPOAb (B = 0.002, *p* = 0.009, OR = 1.002). AUCROC showed the following values for each risk factor: 0.73 for HAMA, 0.679 for TPOAb, and 0.73 for CGI. The combination of HAMA, TPOAb, and CGI was 0.79 (Fig. 2).

**Table 4:** Factors associated with suicide attempts in patients with comorbid dyslipidemia in in mid-adulthood onset

	B	Wald statistic	P	OR	95%CI
HAMA score	0.182	13.13	0.000	1.200	1.090–1.320
CGI	1.022	15.28	0.000	2.778	1.660–4.630
TPOAb	0.002	0.001	0.009	1.002	1.001–1.004

*HAMA* Hamilton anxiety rating scale, *CGI* Clinical global impression, *TPOAb* Thyroid peroxidases antibody

## Discussion

To our knowledge, our study is the first to investigate the frequency and influencing factors of suicide attempts among first-episode untreated patients with MDD with comorbid dyslipidemia in a Chinese Han population. The major discoveries of our study were that the proportion of suicide attempts was higher in both early adulthood-onset and mid-adulthood-onset patients with MDD with comorbid dyslipidemia than in patients with MDD without comorbid dyslipidemia. Among MDD comorbid dyslipidemia, the percentages of suicide attempts reached 22.2% for early adulthood onset and 26.5% for mid-adulthood onset. Suicide attempt factors differed between early and mid-adulthood onset patients with MDD with dyslipidemia.

Few studies have been published on the frequency of suicide attempts in patients with MDD with dyslipidemia. In this study, the prevalence of comorbid dyslipidemia in MDD patients was 61%, which was higher than that in the Han Chinese population without MDD or any psychiatric illness (42.4%) [33]. Also, we observed a higher frequency of suicide attempts in patients with MDD with comorbid dyslipidemia than in those without comorbid dyslipidemia in both early adulthood and mid-adulthood onset. The prevalence of comorbid dyslipidemia is higher in patients with MDD than in the general population for the following reasons: First, patients with MDD have reduced activity and exercise. Some patients have binge eating habits, resulting in an increased proportion of obese and overweight patients [34]. These poor lifestyles eventually lead to abnormal blood lipid levels. Secondly, antidepressants or antipsychotics can affect metabolism, leading to adverse effects such as weight gain and dyslipidemia [35]. However, the patients included in this study were all first-time untreated patients.

Our study found higher levels of TC, LDL, and HDL, but no differences in TG in suicide attempters among patients with MDD with comorbid dyslipidemia. These findings were discovered in patients with MDD onset in both early and mid-adulthood. The relationship between suicide attempts and dyslipidemia has been extensively studied, but the findings are inconsistent. For example, Enrico Capuzzi et al. found that low cholesterol, LDL, and HDL levels were associated with violent suicide [36]. Adrea Aguglia et al. revealed that lower serum cholesterol predicted higher suicide mortality [37]. A meta-analysis reported that lower total cholesterol levels were associated with suicidal ideation, which is partially consistent with the results of the present study [38]. In a Mexican population, Marcela Segoviano-Mendoza et al. identified that relatively low cholesterol levels correlated with suicide among those suffering from MDD [39]. In contrast, the findings of Subin Parker et al. did not provide evidence to support the use of biological indicators such as total serum cholesterol to predict the likelihood of suicide in individuals with severe mental illness [40]. The different results of these studies may be due to differences in the people who took part, the ways they were evaluated, and what was considered dyslipidemia.

In our study, among early-adulthood onset patients with MDD with comorbid dyslipidemia, suicide attempters had higher HAMA scores, CGI scores, and TPOAb levels than non-suicide attempters, which was confirmed by further logistic regression analysis, suggesting that CGI, HAMA, and TPOAb were independent correlates of suicide attempts in patients with early adult-onset MDD with dyslipidemia. Also, we observed that HAMA, CGI, and TPOAb were independent correlates of suicide attempts in patients with mid-adulthood onset patients with MDD with dyslipidemia. Several studies have explored the association between individual anxiety levels and suicide attempts in patients with MDD [41]. Previous studies have consistently concluded that the severity of anxiety in patients with MDD increases suicide attempts. For example, Baek et al. reported that patients with anxious depression were more likely to have more suicidal thoughts and attempts than patients without anxious depression [42]. Based on a WHO mental health survey of national or representative epidemiological interviews in 24 countries, Kessler et al. found that patients with MDD with anxiety symptoms reported higher suicide attempts during the one-year follow-up period [43]. Also, some investigations have demonstrated that patients with MDD with comorbid anxiety are more likely to attempt suicide. Recently, Zhou et al. found that the HAMA score was an independent risk indicator for suicide attempts in patients with MDD with comorbid anxiety [30].

We noted an independent positive association between the CGI score and suicide attempts in patients with MDD with dyslipidemia of both early-adulthood onset and mid-adulthood onset. Gang Ye's study is consistent with our study showing that CGI is an independent risk factor for suicide attempts in patients with MDD [44]. The relationship between CGI and suicide attempts may be explained by the following factors: First, the CGI score represents the severity of the disease [45]. Suicide attempts are one of the basic symptoms that constitute the symptom cluster in patients with MDD [46]. The more pronounced the patient's suicide attempts, suicidal ideation, and suicidal behavior, the higher the CGI score. Second, some researchers have found that anxiety and depression are associated with worse symptoms and more unfavorable treatment outcomes in patients with MDD [41]. Hence, the higher the levels of anxiety and suicidal ideation in patients with MDD, the higher the CGI level.

Our study showed that TPOAb was an independent risk factor for suicide attempts in patients with MDD with dyslipidemia of early- and mid-adulthood onset. Previous studies have also agreed that TPOAb levels in patients with MDD increase suicidality. For example, Shen Yanmei et al. found that TPOAb was associated with a 2.1-fold increased risk of suicide attempts in patients with MDD [47]. Chen S. Wang et al. found that TPOAb was associated with the number of suicide attempts in patients with MDD with glucose dysfunction [48]. The following reasons may explain the correlation between TPOAb and suicide attempts in patients with MDD. First, a population-based survey in Denmark found that TPOAb levels increased the frequency of suicide attempts in the general population [49]. Second, TPO antigens reflect the presence or increased risk of thyroid immune disorders such as Graves' disease and Hashimoto's disease. Michael E et al. noted that autoimmune disorders are a risk factor for mood disorders [50]. Detlef Degner et al. found that TPOAb levels are strongly associated with MDD [51].

In early-adulthood onset patients with MDD with dyslipidemia, three additional factors were independently associated with suicide attempts: suspicion/persecution, systolic blood pressure, and hallucinatory behavior. However, this is not the case for mid-adulthood onset patients with MDD. All of these variances can be explained by the following possible causes: First, a previous study found greater cardiovascular burden in patients with MDD with early adult onset [52]. Dargeld et al. found that emotional hyperreactivity in patients with MDD in early adulthood predicted the risk of suicide in patients with MDD through a machine learning approach [53].Patients who are emotionally hyperactive tend to have higher cardiometabolic disturbances [54]. When they become agitated and attempt suicide, they often exhibit elevated systolic blood pressure but fail to meet the diagnostic criteria for hypertension. Second, an earlier age of onset with psychotic symptoms may be characteristic of bipolar depression [55]. Compared to patients with unipolar depression, patients with bipolar depression are more hyperemotional and more likely to exhibit emotional instability [56]. When patients with bipolar depression are emotionally unstable and have psychotic symptoms such as suspicion and hallucinatory behavior, they are more likely to make suicide attempts [57]. This may partly explain the fact that suspicion/persecution and hallucinatory behavior are independent risk factors for MDD patients with early adult onset, in contrast to MDD with mid-adulthood onset.

The biological mechanisms underlying the increase in suicide attempts in patients with MDD with dyslipidemia are not fully understood. It has been hypothesized that the 5-hydroxytryptamine (5-HT) system may be influenced in some way by lipids. Higher levels of peripheral lipids may alter the viscosity of lipids in neuronal membranes, which in turn affects 5-HT levels [58]. Sequence variants of the 5-HT receptor (HTR5A) have been shown to be associated with high plasma lipid levels [59]. Serotonergic system dysfunction has been extensively linked to suicidal behavior and suicide [60]. Studies of 5HIAA ( a key metabolite of 5HT) in the CSF of suicidal individuals provide the primary evidence establishing the relationship between 5-HT and suicide [61]. According to a meta-analysis, 5-HIAA levels were lower in patients who attempted suicide than in those who did not [62].

Our study has several limitations. First, because this was a cross-sectional survey, we could not clarify the causal relationship between the risk factors and suicide attempts in patients with MDD with combined dyslipidemia. We need well-designed cohort follow-up studies to explore this causal relationship. On the other hand, given that it was a cross-**s**ectional study**,** there was a possibility of recall bias among participants, which may have some impact on the accuracy of the results of this study**.** Second, the patients in our study were all Han Chinese, and the findings lacked extrapolation. Therefore, validation in different countries and ethnic groups is needed. Third, we only used one simple question to assess suicide, but did not use a structured suicide assessment scale, such as the Columbia Suicide Severity Assessment Scale, to assess the severity of suicide in this study. Thus, this approach was susceptible to recall bias and, at the same time, did not measure suicide in a quantitative way. More studies are needed to explore the variables associated with the severity of suicide attempts. Finally, we excluded patients with co-morbid substance use disorders, physical illnesses and other illnesses from this study, which limited the generalizability of the findings in this study.

In conclusion, this study showed that the incidence of suicide attempts was significantly higher in first-episode untreated patients with MDD with dyslipidemia than in those without dyslipidemia. HAMA score, suspicion/persecution, CGI score, TPOAb, systolic blood pressure, and hallucinatory behavior were risk factors for suicide attempts in early-adulthood onset MDD with dyslipidemia. HAMA score, CGI score, and TPOAb were risk factors for suicide attempts in mid-adulthood onset patients with MDD with dyslipidemia. However, due to the limitations of the cross-sectional design and the lack of structured evaluation tools to assess suicide, future studies should use a prospective cohort design to confirm these findings.

## Data Availability

The data that support the findings of this study are available on request from the corresponding author upon reasonable request.

## Abbreviations

ANOVA: Analysis of variance
AUCROC: Area under the receiver operating characteristic
BMI: Body mass index
BP: Blood pressure
CGI: Clinical global impression severity scale
DSM-IV: Diagnostic and statistical manual of mental disorders, Fourth edition
FT3: Free triiodothyronine
FT4: Free thyroxine
HAMD-17: Hamilton depression scale-17 items
HAMA: Hamilton anxiety Scale
HDL: High-density lipoprotein cholesterol
LDL: Low-density lipoprotein cholesterol
MDD: Major depressive disorder
NCEP: National cholesterol education program
NCEP-ATP III: Treatment of high blood cholesterol in adults' final report (NCEP-ATP III)
PANSS: Positive and negative syndrome scale
STAR*D: Sequential treatment alternatives for depression relief (STAR*D)
SCID: Structured clinical interview for DSM-IV (SCID)
SPSS: Statistical package for the social sciences
TC: Total cholesterol
TG: Triglyceride
TgAb: Anti-thyroglobulinand
TPOAb: Thyroid peroxidases antibody
TSH: Thyroid stimulating hormone
